# 1,5-Bis(2-chloro­benzyl­idene)carbonohydrazide

**DOI:** 10.1107/S1600536809026014

**Published:** 2009-07-11

**Authors:** Kaozhen Li, Jing Jiao, Yong Wang, Guo-Dong Wei, Daqi Wang

**Affiliations:** a College of Chemistry and Chemical Engineering, Liaocheng University, Shandong 252059, People’s Republic of China; b Shandong Wuxun High School, Guanxian, Shandong Province 252500, People’s Republic of China; c Shandong Donge Experimental High School, Donge, Shandong Province 252200, People’s Republic of China

## Abstract

In the title mol­ecule, C_15_H_12_Cl_2_N_4_O, the two benzene rings are inclined at a dihedral angle of 14.5 (2)°. In the crystal, inter­molecular N—H⋯O hydrogen bonds link mol­ecules into chains propagated in [001].

## Related literature

For related structures, see: Meyers *et al.* (1995 ▶); Li *et al.* (2008 ▶).
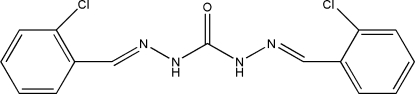


## Experimental

### 

#### Crystal data

C_15_H_12_Cl_2_N_4_O
*M* *_r_* = 335.19
Monoclinic, 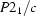

*a* = 10.7889 (11) Å
*b* = 15.7117 (19) Å
*c* = 9.0543 (10) Å
β = 90.978 (1)°
*V* = 1534.6 (3) Å^3^
*Z* = 4
Mo *K*α radiation
μ = 0.43 mm^−1^
*T* = 298 K
0.49 × 0.43 × 0.42 mm

#### Data collection

Bruker SMART APEX CCD area detector diffractometer
Absorption correction: multi-scan (*SADABS*; Sheldrick, 1996 ▶) *T*_min_ = 0.817, *T*_max_ = 0.840
7395 measured reflections
2684 independent reflections
1698 reflections with *I* > 2σ(*I*)
*R*_int_ = 0.048

#### Refinement

*R*[*F* ^2^ > 2σ(*F* ^2^)] = 0.063
*wR*(*F* ^2^) = 0.186
*S* = 1.05
2684 reflections
199 parameters
H-atom parameters constrained
Δρ_max_ = 0.25 e Å^−3^
Δρ_min_ = −0.29 e Å^−3^

### 

Data collection: *SMART* (Siemens, 1996 ▶); cell refinement: *SAINT* (Siemens, 1996 ▶); data reduction: *SAINT*; program(s) used to solve structure: *SHELXS97* (Sheldrick, 2008 ▶); program(s) used to refine structure: *SHELXL97* (Sheldrick, 2008 ▶); molecular graphics: *SHELXTL* (Sheldrick, 2008 ▶); software used to prepare material for publication: *SHELXTL*.

## Supplementary Material

Crystal structure: contains datablocks I, global. DOI: 10.1107/S1600536809026014/cv2581sup1.cif

Structure factors: contains datablocks I. DOI: 10.1107/S1600536809026014/cv2581Isup2.hkl

Additional supplementary materials:  crystallographic information; 3D view; checkCIF report

## Figures and Tables

**Table 1:** Hydrogen-bond geometry (Å, °)

*D*—H⋯*A*	*D*—H	H⋯*A*	*D*⋯*A*	*D*—H⋯*A*
N1—H1⋯O1^i^	0.86	2.15	2.925 (4)	149
N3—H3⋯O1^i^	0.86	2.06	2.863 (4)	154

Symmetry code: (i) 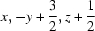.

**Fig. 1.**
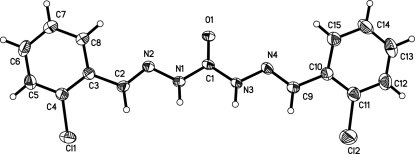
View of (I) showing the atomic numbering scheme and 30% probability displacement ellipsoids.

## supplementary crystallographic information

### Comment

In continuation of our ongoing program directed to the development of environmentally benign methods of chemical synthesis (Li *et al.*, 2008), we present here a user-friendly, solvent-free protocol for the synthesis of substituted carbonohydrazide starting from the fragrant aldehydes and carbohydrazide under solvent-free conditions. Using this method,  we obtained the title compound, (I).

In (I) (Fig. 1), the bond lengths and angles are normal and correspond to those observed in bis(3-fluorophenylmethine)carbonohydrazide (Meyers *et al.*, 1995). Two benzene rings - C3-C8  and C10-C15, respectively - form a dihedral angle of 14.46 (22)°. Intermolecular N—H···O hydrogen bonds (Table 1) link the molecules into chains propagated in direction [001].

### Experimental

*o*-Chlorobenzaldehyde (10 mmol) and carbohydrazide (5.0 mmol) were mixed in 50 ml flash under sovlent-free condtions After stirring 2 h at 373 K, tthe resulting mixture was cooled to room temperature, and recrystalized from ethanol, and afforded the title compound as a crystalline solid. Elemental analysis: calculated for C_15_H_12_Cl_2_N_4_O: C 53.75, H 3.61, N 16.72%; found: C 53.61, H 3.47, N 16.86%.

### Refinement

All H atoms were placed in geometrically idealized positions (N—H 0.86 and C—H 0.93 Å) and treated as riding on their parent atoms, with *U*_iso_(H) = 1.2 *U*_eq_(C) (C, N).

### Crystal data

**Table:**

C_15_H_12_Cl_2_N_4_O	*F*(000) = 688
*M**_r_* = 335.19	*D*_x_ = 1.451 Mg m^−^^3^
Monoclinic, *P*2_1_/*c*	Mo *K*α radiation, λ = 0.71073 Å
*a* = 10.7889 (11) Å	Cell parameters from 2356 reflections
*b* = 15.7117 (19) Å	θ = 2.3–24.5°
*c* = 9.0543 (10) Å	µ = 0.43 mm^−^^1^
β = 90.978 (1)°	*T* = 298 K
*V* = 1534.6 (3) Å^3^	Block, colourless
*Z* = 4	0.49 × 0.43 × 0.42 mm

### Data collection

**Table:**

Bruker SMART APEX CCD area detector diffractometer	2684 independent reflections
Radiation source: fine-focus sealed tube	1698 reflections with *I* > 2σ(*I*)
graphite	*R*_int_ = 0.048
φ and ω scans	θ_max_ = 25.0°, θ_min_ = 1.9°
Absorption correction: multi-scan (*SADABS*; Sheldrick, 1996)	*h* = −12→9
*T*_min_ = 0.817, *T*_max_ = 0.840	*k* = −18→16
7395 measured reflections	*l* = −10→10

### Refinement

**Table:**

Refinement on *F*^2^	Primary atom site location: structure-invariant direct methods
Least-squares matrix: full	Secondary atom site location: difference Fourier map
*R*[*F*^2^ > 2σ(*F*^2^)] = 0.063	Hydrogen site location: inferred from neighbouring sites
*wR*(*F*^2^) = 0.186	H-atom parameters constrained
*S* = 1.05	*w* = 1/[σ^2^(*F*_o_^2^) + (0.078*P*)^2^ + 1.6963*P*] where *P* = (*F*_o_^2^ + 2*F*_c_^2^)/3
2684 reflections	(Δ/σ)_max_ < 0.001
199 parameters	Δρ_max_ = 0.25 e Å^−^^3^
0 restraints	Δρ_min_ = −0.28 e Å^−^^3^

### Special details

**Table:**

Geometry. All e.s.d.'s (except the e.s.d. in the dihedral angle between two l.s. planes) are estimated using the full covariance matrix. The cell e.s.d.'s are taken into account individually in the estimation of e.s.d.'s in distances, angles and torsion angles; correlations between e.s.d.'s in cell parameters are only used when they are defined by crystal symmetry. An approximate (isotropic) treatment of cell e.s.d.'s is used for estimating e.s.d.'s involving l.s. planes.
Refinement. Refinement of *F*^2^ against ALL reflections. The weighted *R*-factor *wR* and goodness of fit *S* are based on *F*^2^, conventional *R*-factors *R* are based on *F*, with *F* set to zero for negative *F*^2^. The threshold expression of *F*^2^ > σ(*F*^2^) is used only for calculating *R*-factors(gt) *etc*. and is not relevant to the choice of reflections for refinement. *R*-factors based on *F*^2^ are statistically about twice as large as those based on *F*, and *R*- factors based on ALL data will be even larger.

### Fractional atomic coordinates and isotropic or equivalent isotropic displacement parameters (Å^2^)

**Table:**

	*x*	*y*	*z*	*U*_iso_*/*U*_eq_	
Cl1	0.51332 (15)	1.04600 (9)	0.84330 (14)	0.0807 (5)	
Cl2	0.80060 (18)	0.37748 (9)	0.60557 (19)	0.0947 (6)	
N1	0.7405 (3)	0.8270 (2)	0.5515 (3)	0.0456 (9)	
H1	0.7471	0.8145	0.6437	0.055*	
N2	0.7084 (3)	0.9075 (2)	0.5094 (3)	0.0423 (8)	
N3	0.7868 (3)	0.6899 (2)	0.5048 (3)	0.0464 (9)	
H3	0.7827	0.6816	0.5985	0.056*	
N4	0.8187 (3)	0.6250 (2)	0.4132 (4)	0.0426 (8)	
O1	0.7578 (3)	0.78083 (18)	0.3143 (3)	0.0475 (8)	
C1	0.7618 (4)	0.7667 (2)	0.4473 (4)	0.0378 (9)	
C2	0.6644 (4)	0.9532 (2)	0.6101 (4)	0.0419 (10)	
H2	0.6528	0.9301	0.7033	0.050*	
C3	0.6312 (4)	1.0421 (2)	0.5826 (4)	0.0395 (10)	
C4	0.5660 (4)	1.0907 (3)	0.6806 (5)	0.0453 (10)	
C5	0.5367 (4)	1.1746 (3)	0.6544 (5)	0.0517 (12)	
H5	0.4927	1.2054	0.7238	0.062*	
C6	0.5721 (5)	1.2122 (3)	0.5267 (6)	0.0613 (13)	
H6	0.5527	1.2688	0.5076	0.074*	
C7	0.6368 (5)	1.1654 (3)	0.4267 (6)	0.0680 (15)	
H7	0.6616	1.1909	0.3393	0.082*	
C8	0.6660 (5)	1.0818 (3)	0.4524 (5)	0.0565 (12)	
H8	0.7096	1.0513	0.3822	0.068*	
C9	0.8362 (4)	0.5526 (3)	0.4735 (5)	0.0437 (10)	
H9	0.8231	0.5459	0.5741	0.052*	
C10	0.8763 (4)	0.4803 (3)	0.3864 (5)	0.0431 (10)	
C11	0.8636 (4)	0.3978 (3)	0.4351 (5)	0.0520 (12)	
C12	0.8999 (5)	0.3289 (3)	0.3534 (6)	0.0650 (14)	
H12	0.8890	0.2739	0.3891	0.078*	
C13	0.9525 (5)	0.3422 (4)	0.2186 (7)	0.0732 (16)	
H13	0.9759	0.2961	0.1609	0.088*	
C14	0.9704 (5)	0.4233 (4)	0.1697 (6)	0.0709 (15)	
H14	1.0089	0.4322	0.0798	0.085*	
C15	0.9330 (4)	0.4914 (3)	0.2500 (5)	0.0571 (12)	
H15	0.9452	0.5461	0.2137	0.069*	

### Atomic displacement parameters (Å^2^)

**Table:**

	*U*^11^	*U*^22^	*U*^33^	*U*^12^	*U*^13^	*U*^23^
Cl1	0.1164 (12)	0.0797 (10)	0.0469 (8)	0.0203 (8)	0.0270 (7)	0.0101 (7)
Cl2	0.1447 (15)	0.0585 (9)	0.0822 (11)	−0.0191 (9)	0.0411 (10)	0.0013 (7)
N1	0.075 (3)	0.042 (2)	0.0196 (17)	0.0078 (17)	−0.0050 (16)	0.0013 (14)
N2	0.061 (2)	0.0383 (19)	0.0277 (19)	0.0013 (16)	−0.0016 (16)	0.0000 (15)
N3	0.079 (3)	0.041 (2)	0.0188 (17)	0.0157 (17)	0.0018 (16)	−0.0016 (14)
N4	0.054 (2)	0.044 (2)	0.0292 (18)	0.0058 (16)	0.0002 (16)	−0.0069 (15)
O1	0.071 (2)	0.0476 (17)	0.0244 (15)	0.0036 (14)	0.0041 (13)	0.0021 (12)
C1	0.047 (2)	0.044 (2)	0.022 (2)	0.0017 (18)	0.0001 (17)	−0.0020 (17)
C2	0.057 (3)	0.039 (2)	0.030 (2)	−0.0028 (19)	−0.0057 (19)	0.0030 (19)
C3	0.044 (2)	0.040 (2)	0.034 (2)	−0.0051 (18)	−0.0049 (18)	−0.0037 (18)
C4	0.055 (3)	0.045 (2)	0.035 (2)	0.000 (2)	−0.0050 (19)	−0.0016 (19)
C5	0.059 (3)	0.044 (3)	0.053 (3)	0.002 (2)	−0.005 (2)	−0.010 (2)
C6	0.079 (4)	0.033 (2)	0.072 (4)	0.001 (2)	−0.007 (3)	0.006 (2)
C7	0.093 (4)	0.050 (3)	0.061 (3)	0.001 (3)	0.022 (3)	0.014 (2)
C8	0.078 (3)	0.043 (3)	0.048 (3)	0.004 (2)	0.013 (2)	0.009 (2)
C9	0.055 (3)	0.046 (2)	0.030 (2)	0.004 (2)	−0.0016 (18)	−0.0003 (19)
C10	0.046 (3)	0.045 (2)	0.039 (2)	0.0059 (19)	−0.0049 (19)	−0.0026 (19)
C11	0.056 (3)	0.045 (3)	0.055 (3)	−0.004 (2)	0.004 (2)	−0.007 (2)
C12	0.075 (3)	0.042 (3)	0.078 (4)	0.001 (2)	0.006 (3)	−0.011 (2)
C13	0.078 (4)	0.062 (3)	0.080 (4)	0.016 (3)	−0.003 (3)	−0.033 (3)
C14	0.076 (4)	0.085 (4)	0.052 (3)	0.019 (3)	0.015 (3)	−0.010 (3)
C15	0.067 (3)	0.061 (3)	0.044 (3)	0.013 (2)	0.000 (2)	−0.002 (2)

### Geometric parameters (Å, °)

**Table:**

Cl1—C4	1.737 (4)	C6—C7	1.367 (7)
Cl2—C11	1.727 (5)	C6—H6	0.9300
N1—C1	1.360 (5)	C7—C8	1.370 (7)
N1—N2	1.364 (5)	C7—H7	0.9300
N1—H1	0.8600	C8—H8	0.9300
N2—C2	1.260 (5)	C9—C10	1.452 (6)
N3—C1	1.339 (5)	C9—H9	0.9300
N3—N4	1.361 (4)	C10—C11	1.377 (6)
N3—H3	0.8600	C10—C15	1.398 (6)
N4—C9	1.275 (5)	C11—C12	1.373 (6)
O1—C1	1.224 (4)	C12—C13	1.371 (8)
C2—C3	1.461 (5)	C12—H12	0.9300
C2—H2	0.9300	C13—C14	1.365 (8)
C3—C4	1.374 (6)	C13—H13	0.9300
C3—C8	1.391 (6)	C14—C15	1.358 (7)
C4—C5	1.374 (6)	C14—H14	0.9300
C5—C6	1.360 (7)	C15—H15	0.9300
C5—H5	0.9300		
			
C1—N1—N2	119.8 (3)	C6—C7—H7	119.3
C1—N1—H1	120.1	C8—C7—H7	119.3
N2—N1—H1	120.1	C7—C8—C3	120.6 (4)
C2—N2—N1	115.0 (3)	C7—C8—H8	119.7
C1—N3—N4	119.3 (3)	C3—C8—H8	119.7
C1—N3—H3	120.4	N4—C9—C10	120.6 (4)
N4—N3—H3	120.4	N4—C9—H9	119.7
C9—N4—N3	116.4 (3)	C10—C9—H9	119.7
O1—C1—N3	123.3 (4)	C11—C10—C15	116.6 (4)
O1—C1—N1	123.5 (4)	C11—C10—C9	122.0 (4)
N3—C1—N1	113.2 (3)	C15—C10—C9	121.5 (4)
N2—C2—C3	121.0 (4)	C12—C11—C10	122.6 (4)
N2—C2—H2	119.5	C12—C11—Cl2	117.1 (4)
C3—C2—H2	119.5	C10—C11—Cl2	120.2 (3)
C4—C3—C8	116.5 (4)	C13—C12—C11	119.1 (5)
C4—C3—C2	123.2 (4)	C13—C12—H12	120.5
C8—C3—C2	120.3 (4)	C11—C12—H12	120.5
C3—C4—C5	122.7 (4)	C14—C13—C12	119.6 (5)
C3—C4—Cl1	120.1 (3)	C14—C13—H13	120.2
C5—C4—Cl1	117.2 (3)	C12—C13—H13	120.2
C6—C5—C4	119.7 (4)	C15—C14—C13	121.1 (5)
C6—C5—H5	120.1	C15—C14—H14	119.5
C4—C5—H5	120.1	C13—C14—H14	119.5
C5—C6—C7	119.0 (4)	C14—C15—C10	120.9 (5)
C5—C6—H6	120.5	C14—C15—H15	119.5
C7—C6—H6	120.5	C10—C15—H15	119.5
C6—C7—C8	121.4 (5)		

### Hydrogen-bond geometry (Å, °)

**Table:**

*D*—H···*A*	*D*—H	H···*A*	*D*···*A*	*D*—H···*A*
N1—H1···O1^i^	0.86	2.15	2.925 (4)	149
N3—H3···O1^i^	0.86	2.06	2.863 (4)	154

Symmetry codes: (i) *x*, −*y*+3/2, *z*+1/2.
